# Conference Report: The FutuRE oF MinimalLy InvasivE GI and Capsule DiagnosTics (REFLECT) Nyborg, Denmark, October 2023

**DOI:** 10.3390/diagnostics14050458

**Published:** 2024-02-20

**Authors:** Ola Selnes, Camilla Thorndal, Lea Østergaard Hansen, Sebastian Radic Eskemose, Anastasios Koulaouzidis

**Affiliations:** 1 Surgical Research Unit, Odense University Hospital, 5700 Svendborg, Denmark; 2 Department of Clinical Research, University of Southern Denmark, 5230 Odense, Denmark; 3 Department of Medicine, OUH Svendborg Sygehus, 5700 Svendborg, Denmark; 4 Department of Social Medicine and Public Health, Pomeranian Medical University, 70-204 Szczecin, Poland

## 1. Introduction

The gastrointestinal (GI) tract, particularly the small bowel (SB), can be challenging for novel investigation tools. Endoscopy, evolving over time as the primary methodology, is now acknowledged as the ‘gold standard’ for most indications of GI investigation, although it only provides a morphological assessment of any mural or luminal lesions in the mucosa. However, we see only what we know, and major elements of the GI tract functions have been ignored reducing the system to just image assessment. Novel investigation tools such as capsule endoscopy (CE) were introduced in the early 2000s and have since emerged as an established option for minimally invasive GI assessment, particularly for the small bowel (SB) [1]. Evolving from the original camera capsules, advancements and technological innovations have diversified these capsules yielding segment-specific camera capsules, sensing capsules, early therapeutic capsules, and robotic capsules.

The fourth symposium “The FutuRE oF MinimalLy InvasivE GI and Capsule DiagnosTics (REFLECT)” convened in Nyborg, Denmark, as a continuing attempt to bring together healthcare professionals, scientists, manufacturers, and experts with a vested interest in the field of minimally invasive GI diagnostics. Attended by the foremost experts in CE and technology, the dissemination of knowledge attained from clinicians, engineers, and the capsule industry led to enlightening discussions and innovative thinking.

This paper seeks to summarize the expert opinions on the past, present, and future of capsule-based technologies. With a specific focus on colon capsule endoscopy (CCE), this paper integrates the essence of 17 lectures and four panel discussions from the annual REFLECT symposium in Nyborg, Denmark, 2023.

## 2. Current Practice of Using Gastrointestinal Capsules

CE is widely accepted as a primary modality for the investigation of the GI tract, although its primary application remains SB diagnostics. However, recent global events, such as the COVID-19 pandemic and the persistent evolution of artificial intelligence (AI), have contributed to the resurgence of colon capsule endoscopy (CCE) through several trials in colorectal cancer (CRC) screening and symptomatic populations [2,3,4]. Although some of these trials are ongoing, several have concluded, and the results are pending with interest, expected in 2024 [5]. Given the high reinvestigation rates for CCEs, identification and subsequent ‘targeting’ of the optimal patient population(s) are pivotal to establishing CCE as a real routine alternative to conventional colonoscopy. Notably, due to its lack of therapeutic capabilities, CCE would suit those with a high pretest probability of a normal (negative) colonoscopy. Recent meta-analyses have confirmed that CCE has a polyp detection equivalent to that of conventional colonoscopy and superior to that of computed tomography colonography (CTC) [6,7,8]. Other studies report high patient acceptability regarding CCE when compared to colonoscopy and CTC (no differences between CCE and OC observed) [9].

However, white-light capsule endoscopes are only one of several capsule applications available for GI investigation [10]. Among the non-white light capsule endoscopes (NWLCEs) already available for market or on trial for market approval is HemoPill (Ovesco Endoscopy AG), designed specifically for the detection of bleeding in the esophagus, stomach, and SB [11]. Others include Medtronic’s SmartPill [12,13], (now withdrawn from the market), and eCelsius (BodyCAP) for continuous core body temperature monitoring [14]. Capsule technologies on trial for market approval include sampling capsules such as the Recoverable Sampling System (RSS) [15], a tool in development as a supplement to OC, and the Atmo Gas Capsule (Atmo Biosciences), with continuous measurements of gases in the GI to provide insights into gut health and microbiome function [16].

## 3. Field Reports of Colon Capsule Endoscopy

Many experts highlighted the pivotal role that a ‘change of heart’ among fellow gastroenterologists, gastrointestinal surgeons, and general practitioners (GPs) is critical in the successful clinical adaption of CCE. GPs serve as a crucial source of information, offering guidance on optimal bowel preparation to enhance patient compliance, elucidating the investigation procedure, and interpreting the potential implications of the identified findings. The involvement and support of GPs are important in reshaping the narrative of CCE investigation for it to change from “investigation with risk of reinvestigation” into a “well-established possibility of avoiding colonoscopy”. Current reinvestigation rates for CCE are around 45–60%, primarily attributed to reported pathology or inadequate bowel preparation [17,18].

In Denmark, Scotland, and England substantial trials aiming to assess the effect of CCE adaption in national health care services are either underway or have been conducted. The ScotCap trial from Scotland, involving over thousands of patients, led to the near-complete national rollout of capsule investigation in all Scottish counties [17]. The pilot study conducted by NHS England included approximately 7000 patients, primarily symptomatic patients referred by GPs with various indications [19]. Both trials underscore the significance of dissemination through primary care and the establishment of predetermined criteria for selecting candidates suitable for CCE investigation. Notably for the purpose of the English pilot study, a training program was introduced for novice capsule readers (not doctors) engaging over 200 nurses as “pre-readers”. This initiative has effectively optimized the utilization of resources and reduced the time spent by doctors per CCE investigation. Findings from NHS England indicate that up to 63% of CCE patients are discharged from the colorectal pathway post-investigation. While the findings from the UK are promising, studies conducted in the Netherlands and France have encountered a lack of patient engagement in CCE, leading to underpowered studies and obstacles in clinical adaption.

Previously conducted and ongoing trials have reported the completion rate of CCE investigations to be approximately 75% [20]. The notable improvement in the CCE completion rate is due to the integration of Prucalopride in bowel preparation protocols. However, further improvements in both bowel preparation and capsule technology, such as battery lifetime, are imperative to achieve more robust completion rates, ideally exceeding 90%. For CCE to attain such high completion rates, new studies should explore optimal target populations and refine the technical aspects. Additionally, addressing various challenges is pivotal for the clinical adoption of CCE. These challenges include conducting economic and pragmatic studies, as well as implementing artificial intelligence (AI) to minimize the interpretation time of the results.

## 4. Technological Advancements in Gastrointestinal Capsules

The future of capsule technology in GI tract investigation is dependent on a fresh approach to drive advanced capsule capabilities. This shift requires close collaboration between engineers, health professionals, and the industry, and forums like REFLECT play a vital role in facilitating this collaboration.

### 4.1. Non-White Light Capsule Endoscopes (NWLCE)

Various GI capsule modalities beyond white-light capsule endoscopes (WLCE) include passive locomotion, sensing, and therapeutic capsules. The capsule modality within the GI tract offers alternatives such as ultrasound and X-ray based pills, prototypes of which have been trialed for clinical application. These capabilities could supplement standard WLCEs in pathology detection. Notably, the HemoPill by Ovesco Endoscopy AG is widely accepted in clinical settings for the detection and localization of GI bleeding. Another NWLCE on trial for market is the Atmo Gas Capsule provided by Atmo Biosciences, introducing a gas-detecting capsule into the diagnostic pathway for GI disorders. However, interpretating volatile biomarkers remains a challenging area with several ongoing trials exploring capsule-supported gas detection as a diagnostic tool for GI disorders. Other capsule inventions exploit the GI microbiome as a biomarker for GI disorders, including CRC. Future interventions might include capsule-administered medication locally in the mucosa.

### 4.2. Artificial Intelligence

The integration of AI in CE covers various focus areas, including localization, completeness of investigation, bowel cleansing quality (CCE only), polyp recognition and characterization, and detection of non-neoplastic disease. In small bowel capsule endoscopy (SBCE), AI has demonstrated high accuracy in detecting erosions and ulcers according to two meta-analyses evaluating SBCE in a combination of computer-assisted/AI investigation [21,22]. The effect of AI in SBCE has shown a significant time reduction of 90%, saving on average 27 min per video/endoscopy [23].

As with most modalities attempting to implement AI, CCE requires multiple annotations of verified polyps for effective deep learning of algorithms. The shortage of large annotated databases presents an obstacle for AI development, prompting data augmentation to resolve this issue. Nevertheless, some studies on AI algorithms trained by augmented data present mixed results. Some experts even claim data augmentation is disadvantageous for polyp detection algorithms due to the potential inclusion of biased annotations in the learning material for data augmentation, which could exaggerate minor annotation biases and lead to significant AI algorithmic errors. The importance of optimal images for training is notable, as quantity is outperformed by quality in the development of AI algorithms. Considerations of difficulties with annotated databases raise the question of CCE’s aim for polyp detection accuracy, as it does not necessarily need to be perfect but simply sufficient.

### 4.3. The Combination of Capsule Endoscopy and Traditional Endoscopy

Magnetic flexible endoscopy, as part of robotic endoscopy, represents a potential future pathway for WLCE. The innovative approach combines dexterity and stability, real-time 3D localization and visualization, including retroflexion of the capsule in all spaces >3 cm. The incorporation of 3D localization facilitates immediate 3D bowel reconstruction, aiding in the identification of missed spots and indicating the high accuracy of this modality for a full investigation yield. These capsules are provided and maneuvered by endoscopists using joysticks through the application of magnetic force for stability. Additional enhancements, such as locally administered water, could further optimize the investigation.

## 5. Clinical Aspects—Early and Late Stage Solutions

The role of colonoscopy as the gold standard in colorectal investigation seems unable to be questioned, primarily due its ability to conduct interventions and its widespread acceptance status as the primary investigation modality. However, during recent discussions, several experts have proposed CCE as an opportunity to alleviate the workload of physicians and health care systems, as traditional colonoscopies necessitate resources and finances. However, substantial work is required to fulfil the requirements of widespread clinical adaption. The evolving AI technology presents a promising pathway for the enhanced detection of mucosal lesions in both colonoscopy and CCE. This advancement indicates a less burdensome future for health professionals while concurrently improving diagnostic accuracy for colon investigations. CCE’s development will, however, provide new insights for colonoscopy if we assume its application will be carried out by fully accredited endoscopists or robots as a triage-based procedure.

The rapid evolution of minimally invasive GI investigation tools will inevitably enter the market and highlights the ongoing trend of over-treatment. Notably, Professor Owen Epstein emphasized the role of colorectal endoscopists (and their innovative tools) as forget the tiny, observe the small, and remove the large, when discussing polyp detection. The current and future expansion of a doctor’s toolbox necessitates cautious consideration, ensuring patient care remains the main priority and that there is realistic evidence.
